# Editorial: Acute: Chronic Workload Ratio: Is There Scientific Evidence?

**DOI:** 10.3389/fphys.2021.669687

**Published:** 2021-05-07

**Authors:** Hassane Zouhal, Daniel Boullosa, Rodrigo Ramirez-Campillo, Ajmol Ali, Urs Granacher

**Affiliations:** ^1^University of Rennes, Department of Sport Sciences. M2S (Laboratoire Mouvement, Sport, Santé) - EA 1274, Rennes, France; ^2^Integrated Institute of Health, Federal University of Mato Grosso do Sul, Campo Grande, Brazil; ^3^Human Performance Laboratory, Department of Physical Activity Sciences, Universidad de Los Lagos, Osorno, Chile; ^4^Facultad de Ciencias, Centro de Investigación en Fisiología del Ejercicio, Universidad Mayor, Santiago, Chile; ^5^School of Sport, Exercise and Nutrition, Massey University, Auckland, New Zealand; ^6^Division of Training and Movement Sciences, University of Potsdam, Potsdam, Germany

**Keywords:** training load, exercise training, injury, global positioning system, internal load, external load

## Introduction

The scientific monitoring of athletes is fundamental to determine and understand the individual biological responses to training (Halson, 2014). In elite sports, it is crucial to regularly monitor training and performance to detect biopositive or negative responses that can be used to effectively program training according to the needs of each athlete (Bourdon et al., 2017). Moreover, workload monitoring can also help to assess fatigue and indicate the need for recovery in different physically demanding situations to ultimately avoid injuries (Halson, 2014). As there is evidence that lower injury rates are associated with higher team sport performances (Eirale et al., 2013), sport scientists and medical staff should regularly and accurately evaluate athletes' injury risk using workload measures (Halson, 2014).

**Figure 1:**
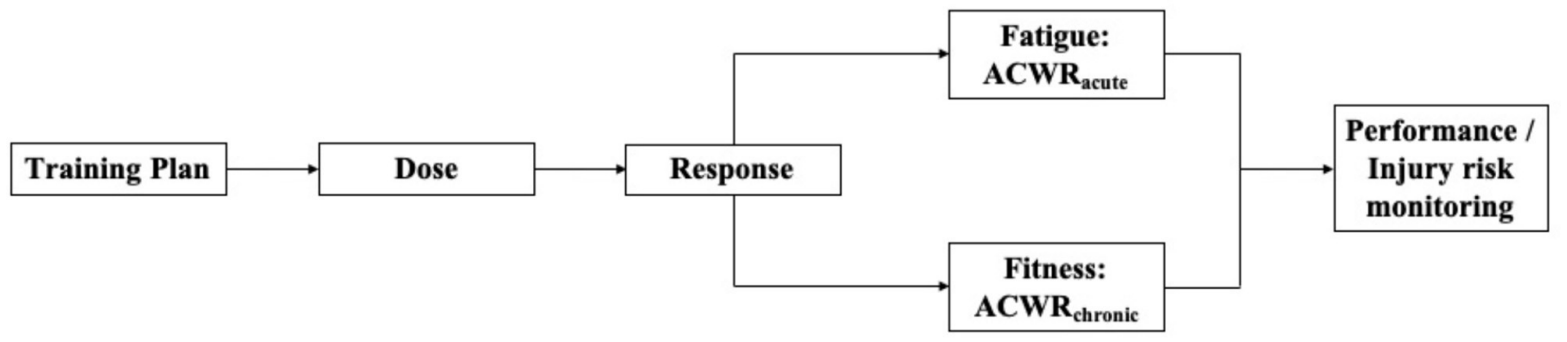
Conceptual model for developing athlete monitoring systems according to the fitness-fatigue model using the acute: chronic workload (ACWR) approach and internal/external workload measures. While the fitness component is comparable to chronic workload (e.g., 28 days rolling average workload), fatigue is comparable to acute workload (e.g., 7 days rolling average workload) (adapted from Coutts et al., 2018).

Based on Banister et al. (1975) fitness and fatigue model, Gabbett et al. (2016) introduced the concept of the acute:chronic workload ratio (ACWR) with acute workload hypothetically representing the fatigue component and chronic workload the fitness component of Banister's model (Figure 1). ACWR allows individualized performance development and injury prevention using the relation between acute to chronic workload data. For this purpose, internal (e.g., heart rate, session-rate of perceived exertion [sRPE] × duration) and external (e.g., performance measures, tracking variables such as running speed and/or acceleration using Global Positioning Systems [GPS]) load measures should be collected to compute ACWR during training and competition (Malone et al., 2017). It has previously been recommended to determine the ratio between acute (training load accumulated during the last 7 days) and chronic (mean training load over the previous 3 to 6 weeks) workloads (Gabbett et al., 2016; Gabbett and Whiteley, 2017). The underlying rationale is that athletes' physical fitness develops adequately if the chronic load progressively increases to high levels while the acute load remains below, similar to, or slightly above the chronic workload (i.e., ACWR range between 0.8 and 1.3). Conversely, the athlete is considered not well-prepared and likely at an increased risk of sustaining acute or overuse injuries if the acute workload exceeds the chronic load (i.e., ACWR ≥ 1.5) (Malone et al., 2017; Windt and Gabbett, 2017).

**Figure 2:**
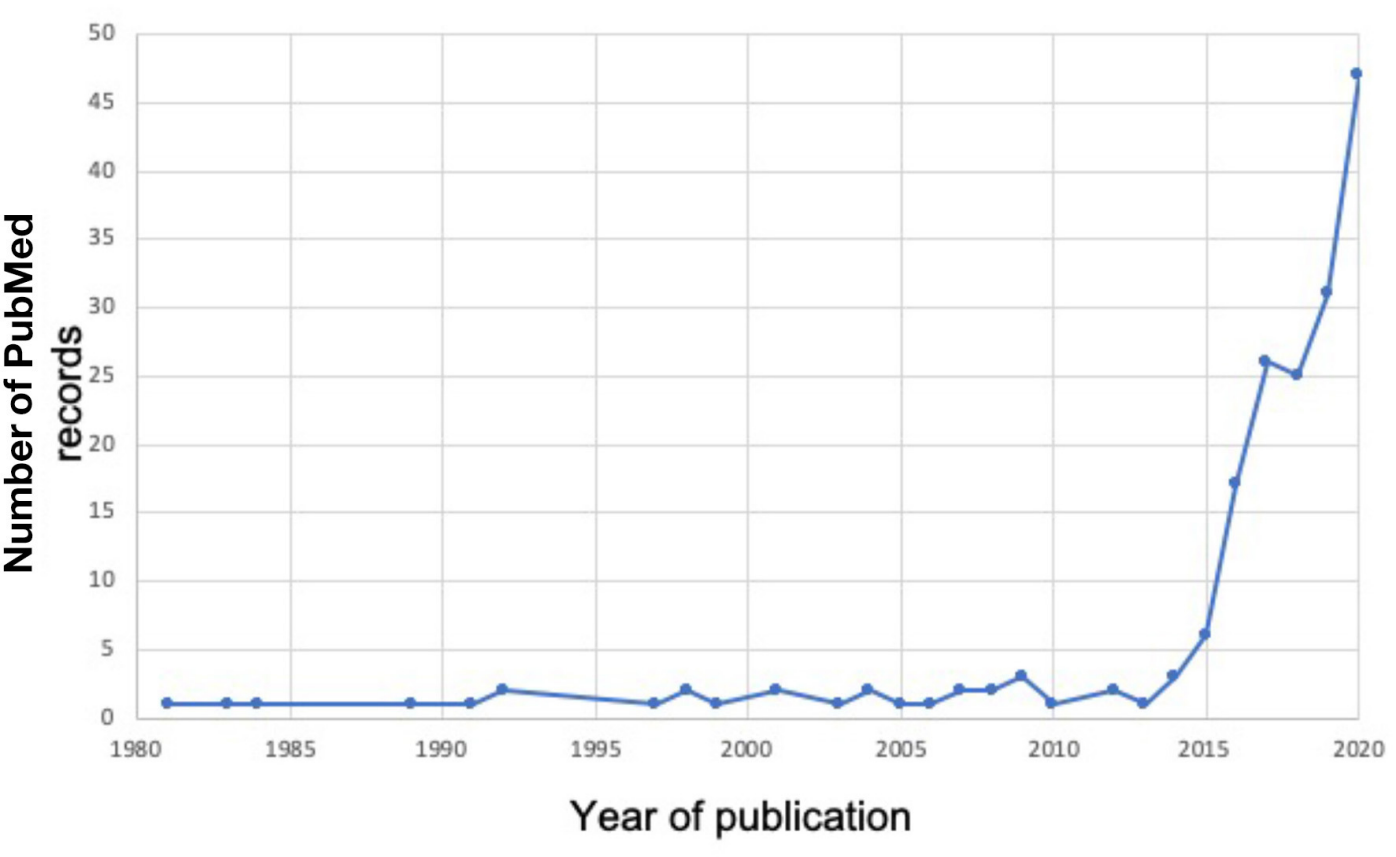
Number of PubMed listed publications per year related to the topic Acute: Chronic Workload Ratio (ACWR) between 1980 and 2020. The following search was applied in the electronic database PubMed (“acute to chronic work load ratio” OR ACWR).

Over the past 15 years, the ACWR approach has received a lot of attention from researchers and practitioners who are active in different sports such as Australian football, cricket, rugby, and soccer (Gabbett, 2016, 2020; Griffin et al., 2020). Figure 2 illustrates the exponential growth in the number of PubMed-listed publications per year on ACWR research using the search syntax (“acute to chronic work load ratio” OR ACWR). In 2016, the International Olympic Committee (IOC) published a consensus statement (Soligard et al., 2016) that suggests the use of the ACWR approach for injury prevention. In addition, Myers et al. (2020) reported level A evidence in support of the sRPE ACWR as an effective tool to prevent non-contact injuries in elite athletes. Despite evidence in favor of the ACWR approach, different authors have raised substantial criticism (Impellizzeri et al., 2020a,b; Wang et al., 2020). For instance, the validity of ACWR has been recently questioned due to the large heterogeneity of the internal (e.g., session RPE, heart rate) and the external load (e.g., GPS data, training time) variables that are used for ACWR calculation (Impellizzeri et al., 2020a,b; Wang et al., 2020). In addition, opponents of the ACWR approach argue that there is no rationale as to the exact time span for acute and chronic workload monitoring (Impellizzeri et al., 2020a,b). In the absence of a rationale, other authors have selected multiple time windows (Delecroix et al., 2018), but again the selected time span appears arbitrary. Another major criticism that has been postulated is that ACWR is a measure of training workload, most often assessed through spatio-temporal metrics from GPS data, but not a mechanical load parameter (Impellizzeri et al., 2020a,b). In a strict biomechanical sense, repetitive mechanical load, but not training load, is associated with an increased risk of tissue damage (Brüggemann and Niehoff, 2011). In other words, training load is not a surrogate of mechanical loading. Overall, the literature on ACWR research is controversial, which is why more research is needed.

Accordingly, it was timely to elucidate strengths and weaknesses of the ACWR approach in the form of a Frontiers Research Topic entitled “**Acute: Chronic Workload Ratio: Is there Scientific Evidence?.”** Thus, the aims of this Research Topic were to provide knowledge on the underlying physiological mechanisms of ACWR and if there is a scientific evidence to support the use of this ratio as an approach for injury risk prediction in different sports.

## Summary of Selected Articles From This Research Topic

Overall, seven articles were published in this Research Topic. The contributing 45 authors are from different countries across the globe including Australia, Brazil, China, Czech-Republic, France, Germany, Iran, Slovakia, Spain, Switzerland, and the United States of America. In terms of content, the seven articles focused on the relation between training workload and diverse biomarkers as well as injury risk. In addition, guidelines were provided for practitioners on how to use and implement ACWR in daily practice.

**Suarez-Arrones et al.**

This study aimed to determine whether spikes in ACWR are associated with injury incidence, and whether the differences in external load are due to high or low match exposure over the course of one soccer season. Fifteen professional soccer players who played for a European Champions League Club were enrolled in this study. External training and match load were assessed from all athletes using GPS. The preliminary results from this study indicate that ACWR spikes are unrelated with subsequent injury occurrence in professional soccer players. Differences in official game exposure caused significant imbalances in the chronic external loads among the team players which should be minimized through individualized training according to match play time.

**Zemková et al.**

These authors performed a scoping review (i) to map the literature that addressed associations between the ACWR and the occurrence of back pain and/or injury in athletes from individual and team sports, and (ii) to identify gaps in the existing literature and propose future lines of research on this topic. The authors concluded that fatigue of the trunk muscles induced by excessive loading of the spine appears to be one of the main sources of back problems in athletes. More specifically, high training volume and repetitive (monotonous) motions are responsible for the observed high prevalence rates. However, limited evidence exists on the relationship between the ACWR and back pain or non-contact back injuries in athletes from individual and team sports.

**Arazi et al.**

These authors aimed to investigate the relationship between the ACWR, based upon the participants' session rating of perceived exertion (sRPE), using two models, (i) rolling averages and (ii) exponentially weighted moving averages, and the injury rate in young male team soccer players aged 17.1 ± 0.7 years during a competitive mesocycle.

The authors concluded that the ACWR using sRPE and training time is easy to administer and useful as a measure to monitor injury occurrence in U-18 male soccer players. A practically relevant finding from this study was that ACWRs calculated with exponential weighting showed stronger correlations with injury occurrence than rolling averages in male team soccer players.

**Sedeaud et al.**

These authors examined the relationship between the occurrence and severity of injuries and three different workload ratios (ACWR, exponentially weighted moving averages, and the robust exponential decreasing index) in elite athletes during one season. This study included elite soccer players and pentathletes and showed significant associations between acute to chronic workload calculations and injury occurrence as well as severity. However, no evidence was found in support of a “sweet spot” ACWR zone (0.8–1.3 zone) that is related to a diminished injury risk.

**Boullosa et al.**

Boullosa et al. pointed out that there are no studies on individual sports (e.g., sports where the athlete competes as an individual rather than in a team) available that prove validity of the ACWR. This could be due to the fact that workload spikes are mostly observed in team sports because of the influence of contextual factors (e.g., several matches, less recovery, environmental factors). For this reason, the authors suggested to individualize training periodization in team sports, as is normally done with individual sports, to avoid excessive fatigue, incomplete recovery or insufficient readiness associated with low fitness.

**Ravé et al.**

In this opinion paper, the authors presented a practical approach for soccer coaches on how to use GPS data for monitoring of training load in team sports using an individualized approach. The authors suggested that the planning of external training load should be realized on a monthly, weekly, and daily level in order to reach collective performance. Within this collective plan, it is important to expose each player to individual external training load to enhance physical performance and lower the risk of sustaining injuries. GPS is a valid, reliable and relevant tool for tracking the external training load in professional soccer. Previous scientific recommendations highlighted the importance of monitoring the external training load to reduce injury risk and optimize players' physical performance. In this opinion paper, the authors proposed an approach on how to use GPS data to analyze, prescribe, and control the external TL in elite soccer, both collectively (i.e., team) and individually. The authors also specified that the application of ACWR may help coaches to get a more detailed insight into each player's training load (TL). This may help to prevent overtraining.

## Conclusions

This Frontiers Research Topic contributes to our understanding of ACWR and it will hopefully stimulate future discussions among researchers and coaches. While the cause-and-effect relationship between training load and injury should be the object of further research, monitoring through simple tools as the ACWR should not be abandoned. In addition, future studies should focus on selected and highly reliable parameters for internal (i.e., session RPE) and external (i.e., GPS data) workload assessment and the exact time span for acute and chronic workload monitoring. More specifically, further research is needed to elucidate if ACWR predicts injury incidence independently of other risk factors. Finally, key physiological parameters should be identified that are associated with ACWR. Knowledge on ACWR-related physiological mechanisms allow to better understand the true predictive potential of ACWR for injury occurrence.

## Author Contributions

All authors listed have made a substantial, direct and intellectual contribution to the work, and approved it for publication.

## Conflict of Interest

The authors declare that the research was conducted in the absence of any commercial or financial relationships that could be construed as a potential conflict of interest.
